# Antidepressant-associated sexual dysfunction in outpatients

**DOI:** 10.1186/s12888-025-06751-1

**Published:** 2025-04-02

**Authors:** Yasir Safak, Sena Inal Azizoglu, Furkan Bahadır Alptekin, Tacettin Kuru, Mehmet Emrah Karadere, Simge Nur Kurt Kaya, Simay Yılmaz, Nisa Nur Yıldırım, Amine Kılıçtutan, Helin Ay, Hüseyin Sehit Burhan

**Affiliations:** 1 Department of Psychiatry, Ankara Etlik City Hospital, Ankara, Turkey; 2 Department of Psychiatry, Basaksehir Cam and Sakura City Hospital, Istanbul, Turkey; 3 Department of Psychiatry, ALKU Alanya Education and Research Hospital, Antalya, Turkey; 4 Department of Psychiatry, Istanbul Medeniyet University, Istanbul, Turkey; 5 Department of Psychiatry, 29 Mayıs State Hospital, Ankara, Turkey

**Keywords:** Antidepressants, Antipsychotic selective serotonin reuptake inhibitors, Depression, Sexual dysfunctions

## Abstract

**Background:**

Antidepressant treatment is related to various sexual dysfunctions. This may cause discontinuation of the medication. This study aims to evaluate the level of sexual dysfunction of antidepressant users and the possible factors linked.

**Methods:**

Demographic variables and sexual dysfunction level of 452 people in total (291 males and 161 females) were assessed by demographic data form and Psychotropic-related Sexual Dysfunction Questionnaire (PreSEXDQ).

**Results:**

Sexual dysfunction was highly prevalent among both females (88.7%) and males (84.5%). Among females, significant differences were observed based on antidepressant type, with those using bupropion experiencing lower levels of sexual dysfunction compared to those on SSRIs, SNRIs, or vortioxetine. In contrast, no significant differences were found for males.

**Conclusions:**

This highlights the importance of considering gender and medication type when addressing and managing psychotropic-related sexual dysfunction. Furthermore, additional studies are needed to determine whether a causal relationship exists between psychiatric medication-related sexual dysfunction and treatment discontinuation.

## Introduction

Antidepressants are associated with various side effects, including potentially worsening sexual function and satisfaction. Sexual dysfunction is one of the most reported side effects of antidepressant use. Many patients encounter disruptions in their sexual function due to the use of these medications [1–4]. Sexual dysfunction, a common side effect of antidepressants, is one of the primary reasons for treatment discontinuation in patients, significantly contributing to non-adherence while remaining one of the most underreported adverse effects [3, 5, 6].

Both depression and antidepressant therapy can adversely affect sexual functioning, with the latter significantly impacting patients’ quality of life [7–9]. Moreover, sexual functioning can influence non-sexual aspects of a relationship, and individuals in sexually inactive marriages tend to have lower levels of marital happiness [10, 11]. Antidepressant-related sexual dysfunction can affect all dimensions of sexual activity, including desire, arousal, and orgasm, for both males and females. Females frequently encounter challenges with sexual desire, arousal, and orgasm while using antidepressants, whereas males experience issues primarily with desire and orgasm [3, 12].

The use of antidepressant treatment is linked to sexual dysfunction (SD) at rates of up to 70%, with some variation associated with different medicines within this class [8]. Selective serotonin reuptake inhibitors and serotonin noradrenaline reuptake inhibitors with strong serotonergic activity have a higher risk of sexual dysfunction than some other antidepressants, such as tricyclic antidepressants, except clomipramine, Monoamine oxidase inhibitors, vortioxetine, mirtazapine, and bupropion. Sexual dysfunction induced by selective serotonin reuptake inhibitors (SSRIs) can reach rates of up to 80% [3, 4, 13]. Serotonin, as the primary neurotransmitter, may play a role in affecting sexual function negatively. The postsynaptic serotonin 2 A receptor activation can significantly contribute to antidepressant-induced sexual dysfunction. Among the various SSRIs, paroxetine has been associated with a higher risk of sexual dysfunction than others in its class [14]. Paroxetine may cause higher rates of sexual side effects due to its mechanism of action, as it blocks D2 receptors, which are directly involved in sexual functions, and inhibits the synthesis of nitric oxide, which is essential for erections [1]. With SSRIs or serotonin noradrenaline reuptake inhibitors (SNRIs), 27–65% of female patients and 26–57% of male patients experienced either a deterioration of pre-existing difficulties or the onset of new sexual problems during the initial weeks of treatment [4]. The occurrence of “treatment-emergent sexual dysfunction” was not more prevalent with antidepressants such as agomelatine, bupropion, and mirtazapine than with placebo. Bupropion demonstrated a significantly lower rate of treatment-emergent sexual dysfunction compared to SSRIs like escitalopram, fluoxetine, paroxetine, or sertraline. This difference may be attributed to bupropion’s mechanism of action, which predominantly affects noradrenaline and dopamine [3, 4, 15]. Mirtazapine was found to be less likely to cause adverse sexual effects compared to other antidepressants [1]. Vortioxetine, a novel antidepressant, was associated with a low incidence of reported adverse effects on sexual function in both males and females [4, 16].

Patients often hesitate to discuss such issues with their treating physicians spontaneously. This reluctance is supported by findings from multiple studies that compare the rates of spontaneously reported sexual dysfunction with rates of reporting only after specific questioning [8]. The reporting of sexual dysfunction is likely underestimated due to various factors. Patients and clinicians may attribute sexual dysfunction associated with medication to other causes, such as linking the effects to relationship issues or psychopathology rather than the medication itself. Additionally, the act of spontaneously reporting sexual side effects can be challenging as it may be considered shameful or embarrassing. Consequently, expressing these concerns after specific questioning may be more feasible and informative [10, 13]. In a study involving 344 patients receiving SSRIs, it was observed that the incidence of sexual dysfunction was significantly higher (58%) when physicians directly asked patients about it compared to cases where patients spontaneously reported sexual dysfunction [17].

The current research aims to assess the prevalence of reported sexual dysfunction among individuals using antidepressant medication and examine the relationship between sexual dysfunction level, gender, age, marital status, and the antidepressant being used. This study hypothesizes that antidepressants with serotonergic activity, such as SSRIs and SNRIs, are linked to higher SD levels compared to non-serotonergic agents like bupropion and mirtazapine.

## Methods

### Participants

The non-random convenience sampling method has been used due to time benefits. All participants selected from the Psychiatry Outpatient Clinic of four different hospitals from three major cities (Istanbul, Ankara, Antalya) in Türkiye between January 2023 and January 2024 still used antidepressant monotherapy for at least one month and volunteered to participate in research. The people were informed about the study and filled out the demographic form and PRSexDQ. Five hundred fourteen people volunteered to participate in the study. The study collected data from participants at four hospitals in three major cities in Türkiye (Ankara, Antalya, and İstanbul). Since 58 of these people used more than one psychiatric drug, they were not included in the analysis. Data from 4 people using mirtazapine, trazodone, or quetiapine also were not included in the analysis because the numbers for the cell would not be sufficient. Analyzes were made using data obtained from 452 people.

### Psychometric measurements tools

Demographic Data Form: For this study, researchers designed a self-report form with questions regarding participants’ age, gender, marital status, and treatment histories. Psychiatric diagnoses were determined based on clinical records and patient history.

Psychotropic-related Sexual Dysfunction Questionnaire: The self-report form is administered through a clinical interview. The first two items assess whether there have been any changes in sexual activity linked to Antidepressant treatment. The remaining five items rate the intensity or frequency of changes in sexual function. These items are about decreased libido, delay in orgasm-ejaculation, lack of orgasm-ejaculation, erectile dysfunction in men, impaired vaginal lubrication in females, and the patient’s tolerance of sexual dysfunction. Tolerance refers to the degree to which patients can endure or accept the changes in their sexual function without significant distress. This item asks how well patients could cope with or adapt to these changes. The scale’s total score can be obtained by summing the scores of items 3–7. The total score is as follows: no sexual dysfunction: 0, mild: 1–5, moderate: 6–10 or any item = 2, severe: 11–15, or any item = 3 [15]. A study on Turkish validity and reliability is available. The reliability analysis revealed a Cronbach’s Alpha coefficient of 0.906. It has been shown to correlate with the Arizona Sexual Experience Scale and the Golombok Rust Inventory of Sexual Satisfaction, indicating that, due to its psychometric properties, PReSexQD can effectively measure both sexual dysfunction and sexual satisfaction [18].

### Statistical analysis

The data was analyzed using Jamovi (version 2.3.21.0) [19]. Mean, median, standard deviation (SD), and interquartile range (IQR) were used to present demographic and clinical characteristics for continuous variables, and frequency and percentage were used for categorical variables. Pearson and Spearman correlation analyses were employed to explore relationships among constant variables. Differences between groups were assessed using Kruskal-Wallis, and pairwise comparisons were evaluated using the Dwass-Steel-Critchlow-Fligner method.

## Results

### Descriptive statistics for demographic and clinical data

The ages of the participants ranged from 18 to 69 (Mean = 38.0, SD = 9.96). Of the participants, 291 (64.4%) were female, and 161 (35.6%) were male. There is no significant difference between the mean age of females and males on the independent-sample t-test (Mean = 37.3, SD = 8.97; Mean = 39.1, SD = 11.5; *p* =.069). Three hundred and fifty-five (78.5%) of the total participants, 241 (82.8%) of the females, and 114 (70.8%) of the males were married. The chi-square test shows a significant difference in marital status between females and males (*p* =.012). There is a significant difference in diagnosis and medications between females and males in the chi-square test (*p* =.179, *p* =.802).

The data provided a diagnosis for 452 people; depression was the most prevalent condition, with 183 cases (46.0%). Anxiety followed closely behind, with 157 (39.4%). Obsessive-Compulsive Disorder (OCD) had 18 (4.5%). Depression and Anxiety represented 37 (9.3%) of the total. Attention deficit and hyperactivity disorder (ADHD) and somatization disorder each had 1 case (0.3%). All 452 participants provided data for medication. A hundred and twenty-two (27.0%) individuals were using sertraline, escitalopram with 100 individuals (22.1%), fluoxetine with 85 individuals (18.8%), paroxetine with 56 individuals (12.4%), citalopram and bupropion each with 12 individuals (2.7%), venlafaxine with 20 individuals (4.4%), Duloxetine with 25 individuals (5.5%), and vortioxetine with 20 individuals (4.4%). Diagnoses and medications for males and females are listed separately in Table 1. According to PReSexQD, 258 (88.7%) of females and 136 (84.5%) of males reported experiencing some degree (mild, moderate, or severe) of sexual dysfunction (Table 1).

**Table 1:** Descriptive statistics for demographic and clinical variables

	Females *n* = 291	Males *n* = 161	*p*
Ages *Mean (SD)*	37.3, 8.97	39.1, 11.5	.069^a^
Married *n (%)*	241 (82.8%)	114 (%70.8%)	.012^b^
Diagnoses *n (%)*			.179^b^
Depressive disorders	153 (58.8%)	67 (48.6%)	
Anxiety Disorders	94 (36.2%)	64 (46.4%)	
Obsessive-Compulsive and Related Disorders	12 (4.6%)	5 (3.6%)	
Somatic Symptom and Related Disorders	0	1 (0.7%)	
Attention Deficit Hyperactivity Disorder	1 (0.4%)	1 (0.7%)	
Medication *n (%)*			.802^b^
SSRI	243 (83.5%)	132 (82.0%)	
Sertraline	76 (26.1%)	46 (28.6%)	
Escitalopram	64 (22.0%)	36 (22.4%)	
Fluoxetine	62 (21.3%)	23 (14.3%)	
Citalopram	7 (2.4%)	5 (3.1%)	
Paroxetine	34 (11.7%)	22 (13.7%)	
SNRI	30 (10.3%)	15 (9.3%)	
Venlafaxine	13 (4.5%)	7 (4.3%)	
Duloxetine	13 (4.5%)	8 (5.0%)	
Vortioxetine	11 (3.8%)	9 (5.6%)	
Bupropion	7 (2.4%)	5 (3.1%)	

^a^ Student t-test, ^b^ Chi-square test

SD = standard deviation, SSRI = Selective serotonin reuptake inhibitors, SNRI = serotonin and norepinephrine reuptake inhibitors

### Statistics for PRSexDQ for females and males

56.9% of the Females responded positively to the first PRSexDQ question (Have you observed any change in your sexual activity since you began taking the treatment? ), totaling 165 individuals. 100 (%34.5) of female participants spontaneously disclosed subjective sexual perceptions without the need for specific questioning to identify sexual dysfunction. Sexual dysfunction was found to be absent in 33 (11.3%), mild in 68 (23.4%), moderate in 86 (29.6%), and severe in 104 (35.7%) of females, according to PRSexDQ scores. A positive correlation was not found between age and PRSexDQ score in females (*p* =.056). Descriptive statistics for females and males on PreSexQD are listed in Table 2.

60.9% of the males responded positively to the first question of PRSexDQ, totaling 98 individuals. 68 (42.5%) of male participants spontaneously disclosed subjective sexual perceptions without the need for specific questioning to identify sexual dysfunction. Sexual dysfunction was found to be absent in 25 (15.5%), mild in 47 (29.2%), moderate in 64 (39.8%), and severe in 25 (15.5%) of men, according to PRSexDQ scores. A positive correlation (*r* =.234) was found between age and PRSexDQ score, indicating that sexual dysfunction severity increases with age for males in Pearson correlation analysis (*p* =.003).

**Table 2:** Descriptive statistics for in females and males on PreSexQD

	Females		Males	
	Median	IQR	Median	IQR
SSRI	6.00	3.00, 10.00	4.00	1.75, 8.00
Sertraline	5.00	2.00, 10.00	4.00	2.00, 8.00
Escitalopram	6.00	4.00, 10.00	3.00	1.00, 7.25
Fluoxetine	7.00	3.00, 11.00	4.00	1.50, 6.50
Paroxetine	7.00	3.00, 9.00	5.00	1.50, 7.75
Citalopram	6.00	4.00, 11.50	5.00	5.00, 6.00
SNRI	7.00	3.00, 10.00	4.00	1.00, 7.50
Venlafaxine	7.00	2.00, 10.00	4.00	1.00, 7.50
Duloxetine	8.00	2.00, 11.00	3.50	0.75, 5.00
Vortioxetine	9.00	7.50, 9.50	5.00	4.00, 11.00
Bupropion	1.00	0.00, 2.00	2.00	0.00, 2.00

IQR = Interquantile Range at %25 and %75, SSRI = Selective serotonin reuptake inhibitors, SNRI = serotonin and norepinephrine reuptake inhibitors

No significant difference was found when comparing the responses to questions first and second question of PRSexDQ between male and female genders using a chi-square test (*p* =.414, *p* =.192).

### Comparison of the level of sexual dysfunction according to the type of antidepressants on the PRSexDQ

The relationship between medication used and sexual dysfunction was evaluated separately for females and males. The Kruskal-Wallis test was chosen because the data does not follow a normal distribution [20]. According to the Kruskal Wallis test, there was a significant difference in sexual dysfunction levels in females depending on the type of antidepressants used (*p* =.006). In pairwise comparison, The PreSexDQ scores in females using bupropion were found to be significantly lower compared to females using SSRIs, SNRIs, or vortioxetine (*p* =.007, *p* =.027, *p* =.003). A similar difference was not significant for males (*p* =.084) (Tables 3 and 4).

**Table 3:** Pairwise comparison of the level of sexual dysfunction according to the type of antidepressants for females

	SSRI	SNRI	Vortioxetine	Bupropion
SSRI				
SNRI	999			
Vortioxetine	480	815		
Bupropion	**007**	**032**	**003**	

Bold values ​​are statistically significant

SSRI = Selective serotonin reuptake inhibitors, SNRI = serotonin and norepinephrine reuptake inhibitors

**Table 4:** Pairwise comparison of the level of sexual dysfunction according to the type of antidepressants for males

	SSRI	SNRI	Vortioxetine	Bupropion
SSRI				
SNRI	0.910			
Vortioxetine	0.725	0.588		
Bupropion	0.118	0.462	0.060	

*p*-values for Dwass-Steel-Critchlow-Fligner pairwise comparison, Bold values ​​are statistically significant. SSRI = Selective serotonin reuptake inhibitors, SNRI = serotonin and norepinephrine reuptake inhibitors

### Comparison of the level of sexual dysfunction according to the type of SSRI on the PRSexDQ

The relationship between the type of SSRI used, and sexual dysfunction was evaluated separately for females and males. According to the Kruskal Wallis test, there was a significant difference in sexual dysfunction levels in both females and males depending on the type of SSRI used (*p* =.583, *p* =.605) (Tables 5 and 6).

**Table 5:** Pairwise comparison of the level of sexual dysfunction according to the type of SSRI for females

	Sertraline	Escitalopram	Fluoxetine	Paroxetine	Citalopram
Sertraline					
Escitalopram	0.628				
Fluoxetine	0.603	1.000			
Paroxetine	0.751	1.000	1.000		
Citalopram	0.928	0.999	0.999	0.998	

*p*-values for Dwass-Steel-Critchlow-Fligner pairwise comparison, Bold values ​​are statistically significant

**Table 6:** Pairwise comparison of the level of sexual dysfunction according to the type of SSRI for males

	Sertraline	Escitalopram	Fluoxetine	Paroxetine	Citalopram
Sertraline					
Escitalopram	0.928				
Fluoxetine	0.957	1.000			
Paroxetine	0.996	0.855	0.892		
Citalopram	0.882	0.625	0.798	0.994	

*p*-values for Dwass-Steel-Critchlow-Fligner pairwise comparison, Bold values ​​are statistically significant

### Relationship between drug dosage and sexual dysfunction

The study examined the relationship between each of the eight antidepressants’ dosages and PreSexDQ scores using Spearman correlation analysis. No relationship was found between dosage and side effects for any of the medications (*p* >.05).

## Discussion

The study, which included data from 452 participants and monitored for sexual problems with PRSexDQ, found that a high percentage of both males and females reported sexual dysfunction. According to the results of the current study, 88.7% of females and 84.5% of males reported experiencing some degree of sexual dysfunction. These results are consistent with those previously reported. Several studies have reported a high prevalence of sexual dysfunction, reaching up to 70–80%, across various antidepressants; this rate appears to increase further with SSRIs and SNRIs in particular [3, 5, 16]. The fact that 93% of the sample of this study is undergoing treatment with SSRIs or SNRIs also supports the high rates of sexual dysfunction observed.

The pathophysiology of sexual dysfunction is complex and not fully understood. However, serotonin, noradrenaline, dopamine, and acetylcholine can affect the central nervous and genital systems. Some papers stated serotonin may be one of the neurotransmitters with adverse effects on sex drive and function, affecting both central and peripheral systems. The activation of post-synaptic serotonin 2 A receptors in the central serotonergic system significantly contributes to antidepressant-induced sexual dysfunction. Conversely, dopamine and noradrenaline agonists may positively affect sex drive and arousal. Considering that the mesolimbic dopaminergic system is believed to play a significant role in maintaining sexual desire and arousal [3, 8]. Antidepressant-Induced Sexual Dysfunctions might be related to the overactivation of post-synaptic 5-hydroxytryptamine (5-HT) 2 A receptors in the raphe nuclei of the midbrain [21]. Among SNRIs, venlafaxine has the most sexual side effects due to its potent serotonin reuptake inhibition [9]. Treatment with vortioxetine may be associated with a low incidence of reported adverse effects on sexual function in males and females. This may be partly due to its antagonist effects at the 5-HT3 receptor and its indirect effects in increasing dopamine and noradrenaline availability [4]. Bupropion, a norepinephrine and dopamine reuptake inhibitor, does not affect the serotonergic system but instead acts on dopamine and norepinephrine transporters, enhancing monoaminergic function by inhibiting the reuptake of these two neurotransmitters. The lack of action on serotonin is believed to be why bupropion is associated with a lower incidence of sexual dysfunction [22]. In recent reviews, vortioxetine showed no significant difference in treatment-emergent sexual dysfunction compared to placebo and is generally well tolerated. However, it should be noted that vortioxetine at 20 mg was associated with numerically lower levels of sexual dysfunction than paroxetine, but this difference did not reach statistical significance. It is associated with increased sexual dysfunction at higher doses (10 to 20 mg/daily), indicating a medium to high risk of sexual dysfunction [9, 10, 23].

According to the results, the only group with differing levels of sexual dysfunction was females using bupropion. The PRSexDQ scores for females using bupropion were significantly lower than those for females using SSRIs, SNRIs, or vortioxetine. For males, the levels of sexual dysfunction measured by PRSexDQ scores did not differ between groups according to the class of antidepressants used. As mentioned above, studies have indicated that bupropion has a lower impact on sexual functioning [3, 4, 10, 22].

Vortioxetine blocks the serotonin transporter and has a strong affinity for several serotonergic receptors: it antagonizes 5-HT3 and 5-HT7 receptors, partially agonizes 5-HT1B, and fully agonizes 5-HT1A. This combined action increases extracellular serotonin, dopamine, and noradrenaline. The risk of sexual dysfunction with vortioxetine increases at the highest doses [10, 23]. In the sample of the current study, no difference in the level of sexual dysfunction was found between the groups using vortioxetine and those using SSRIs and SNRIs for both males and females. The recommended dose for vortioxetine is 10 mg/day [24]. In our sample, the average dose for individuals using vortioxetine was found to be relatively high, at 13.3 mg for females and 11.9 mg for men.

The likelihood of experiencing sexual dysfunction symptoms after antidepressant treatment varies across different classes of antidepressants and, more specifically, among medications within the same class. Serotonergic antidepressants, including SSRIs, SNRIs, and clomipramine, are all associated with a high frequency of sexual dysfunction, with no significant differences among individual drugs. In contrast, non-serotonergic drugs such as bupropion, mirtazapine, and agomelatine have the lowest rates of sexual dysfunction [1, 3, 13, 16]. Neuroimaging studies show that SSRIs cause sexual dysfunction by affecting the central and, to a lesser extent, the peripheral nervous systems, likely due to decreased dopamine in the mesolimbic pathway and suppression of spinal ejaculatory centers. SNRIs impact sexual function based on the balance of norepinephrine versus serotonin reuptake inhibition. Venlafaxine, a potent serotonin reuptake inhibitor, has the highest sexual side effects among SNRIs. Meta-analyses report sexual dysfunction rates of 70–80% with SSRIs and 45% with SNRIs and escitalopram [9]. In this study, in partial agreement with the results mentioned in previous studies, it was found that females using bupropion experienced less sexual dysfunction compared to those using SSRIs, SNRIs, and vortioxetine, which have prominent serotonergic effects.

No significant differences were found in the levels of sexual dysfunction scores among various SSRIs. However, interpreting significant variance with small sample sizes, such as 10 participants per SSRI in this study, can be challenging. Previous research indicated no differences between SSRIs except for paroxetine, which was associated with increased sexual dysfunction, likely due to its potent serotonergic effects [2, 11]. Larger-scale studies are necessary to comprehensively compare sexual side-effect levels across different types of SSRIs, as they may provide more precise insights into their comparative effects on sexual function.

In a study, 41% of patients spontaneously reported sexual dysfunction, with males reporting it more frequently than females. Males may have a lower tolerance for this side effect and are likelier to report it spontaneously than females [16]. In another study, gender did not predict the presence or severity of sexual dysfunction. However, it was found that females reported antidepressant-related sexual dysfunction less frequently and that it had a lesser impact on their tolerability of the medication [16]. Also, differences in sexual physiology and related sexual issues between genders make comparisons challenging, and sexual dysfunction in females has often been overlooked and underreported [25]. In this study, 34.5% of female participants and 42.5% of male participants spontaneously disclosed subjective sexual perceptions, indicating differing levels of self-reported sexual dysfunction between genders. Similar rates of spontaneous reporting of sexual dysfunction were found in the mentioned study; however, despite higher rates in males, no significant difference was found between males and females. Increasing the sample size may increase the likelihood of the possible difference becoming statistically significant. There was no positive correlation between age and PRSexDQ score in females, whereas a positive correlation was found between age and PRSexDQ score in males. It has been reported that the incidence of sexual dysfunction associated with antidepressant medications tends to increase with older age [16, 26]. Sexual dysfunction is related to medical problems that can become more prevalent with age. While aging and functional decline can impact sexual function, physicians should rule out underlying diseases or medication side effects when diagnosing sexual dysfunction [27].

### Limitations

This study has several limitations that should be acknowledged. Firstly, the cross-sectional study design poses a significant limitation in establishing a causal relationship between potential factors and sexual dysfunction. Since this design captures data at a single point in time, it cannot determine whether the observed sexual dysfunction is a direct consequence of the factors being studied, such as antidepressant use, or if other unmeasured variables are influencing the results. Additionally, this design is inadequate for examining the effects of medication changes, dose adjustments, or treatment discontinuation on sexual dysfunction. This limitation underscores the need for longitudinal studies, which can provide a clearer understanding of causality by tracking changes in sexual function over time concerning these specific factors.

This study did not include specific measures to evaluate the impact of relationship or mental health issues on sexual dysfunction, which may result in an incomplete understanding of the factors influencing the observed outcomes. Additionally, no data was available on variables that may impact sexual dysfunction, such as medical status and some other psychological factors. Another limitation is that there may be a relatively low sample size; further statistical analyses with a larger sample size may yield significant results. The lack of significant results regarding the relationship between the type of antidepressant used in males and sexual dysfunction may be related to the low sample size. The absence of a control group is also a limitation. Due to their infrequent use as monotherapy and, therefore, not being included in the statistics, the relationship between sexual dysfunction and medications such as mirtazapine, trazodone, agomelatine, and quetiapine could not be evaluated. The use of different medications from various groups and varying doses has made it difficult to compare the effects. The Ambrosetti et al. study emphasizes the increased severity of psychiatric conditions during and after the COVID-19 lockdown [28]. However, the data collected reflects a period when stressors from the later stages of the pandemic had relatively diminished, potentially overlooking the pandemic’s broader impact. This limitation could still be acknowledged as a factor in the study.

## Conclusion

Given the high rates of depression and antidepressant use, antidepressant-related sexual dysfunction emerges as a significant public health concern. It adversely affects self-esteem, quality of life, mood, and partner relationships [7, 8, 10, 11]. Sexual side effects typically manifest early during antidepressant therapy, and crucially, it often leads to non-compliance with treatment, as both males and females may discontinue medication due to perceived sexual side effects [1, 3]. Healthcare providers should routinely monitor sexual dysfunction in patients on antidepressants, especially during medication changes, and discuss potential side effects when selecting treatment [9, 16]. Spontaneous reporting of sexual side effects often underestimates their prevalence; instead, reliable and sensitive rating scales should be used for accurate assessment [4]. The current study provides valuable insights into the frequency of sexual dysfunction among antidepressant users seeking psychiatric care in major Turkish cities, addressing a notable research gap. It highlights the prevalence and characteristics of sexual dysfunction as a significant side effect of antidepressant treatment, which may often be underreported in clinical settings. This study has the potential to yield substantial data regarding the sexual side effects associated with antidepressant treatment in Turkey. Furthermore, the study underscores the need for heightened awareness and proactive assessment of sexual side effects in psychiatric practice, particularly considering the relatively high rates of sexual side effects, to ensure comprehensive care for individuals with mental health disorders. Examining this issue substantially contributes helpful data to enhance mental health treatment strategies in Türkiye.

## Data Availability

The datasets used and/or analysed during the current study are available from the corresponding author on reasonable request.

## Abbreviations

PreSEXDQ: Psychotropic-related Sexual Dysfunction Questionnaire
SD: Sexual dysfunction
SSRIs: Selective serotonin reuptake inhibitors
SNRIs: Serotonin noradrenaline reuptake inhibitors
SD: Standard deviation
IQR: Interquartile range
OCD: Obsessive-Compulsive Disorder
ADHD: Attention deficit and hyperactivity disorder
5-HT: 5-hydroxytryptamine
